# Supplementary material for: The transcription factor Slug represses p16Ink4a and regulates murine muscle stem cell aging
Source: Nat Commun. 2019 Jun 12;10:2568. doi: 10.1038/s41467-019-10479-4 (PMC6561969; doi:10.1038/s41467-019-10479-4)
Supplement: Supplementary file 1 — Supplementary Information [file 41467_2019_10479_MOESM1_ESM.pdf]

SUPPLEMENTARY INFORMATION

The transcription factor Slug represses p16Ink4a and regulates murine muscle stem cell aging

Zhu et al.

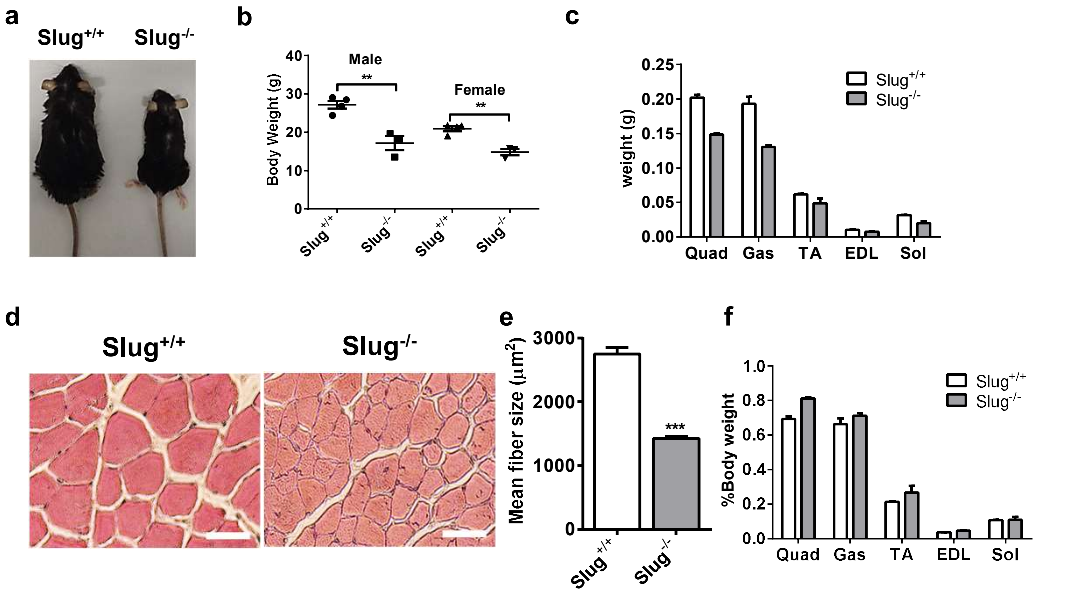

Supplementary Figure 1. Slug knockout mice were abnormal in body size, weight and skeletal muscle.

a. Physical appearance of adult *Slug*$^{+/+}$ and *Slug*$^{-/-}$ mice.
b. The body weights of male and female adult Slug$^{-/-}$ mice and their corresponding wild-type mice (n = 3-4 mice per genotype) **p < 0.01 by Student's *t* test.
c. Comparison of the weights of hindlimb muscles between adult *Slug*$^{-/-}$ and wild-type mice. Quad, quadricep; Gas, gastrocnemius; TA, tibialis anterior; EDL, extensor digitorum longus; Sol, soleus.
d. Representative H&E staining of the intact TA muscles in *Slug*$^{+/+}$ and *Slug*$^{-/-}$ mice (n = 3 mice per group). Scale bar, 50 μm.
e. Quantification of the mean myofiber cross-sectional area (CSA, μm^2) of the intact TA muscles in (**d**).
f. The weights of different hindlimb muscles were normalized to body weight.

Source data are provided as a Source Data file.

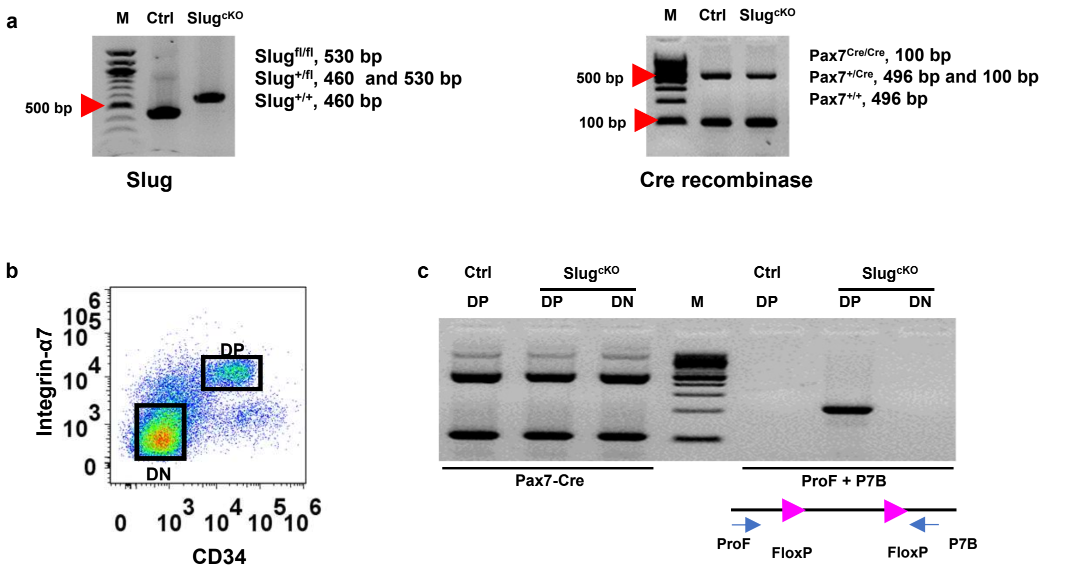

Supplementary Figure 2. Genotyping PCR confirming conditional knockout of Slug in SCs.

a. Genotyping PCR amplifying Slug and Cre recombinase in tail lysates. Ctrl refers to a genotype of Slug$^{+/+}$Pax7$^{Cre/+}$, Slug^{cKO} refers to a genotype of Slug$^{fl/fl}$Pax7$^{Cre/+}$.
b. Representative flow cytometry plots for gaing MuSCs (DP, integrin-α7^{+}CD34^{+}) and non-MuSCs (DN, integrin-α7^{-}CD34^{-}) in CD45^{-}CD11b^{-}CD31^{-}Sca1^{-} subpopulation.
c. Genotyping PCR amplifying Cre recombinase and Slug in SCs (MuSCs) and non-MuSCs lysates. M, 100-bp DNA ladder.

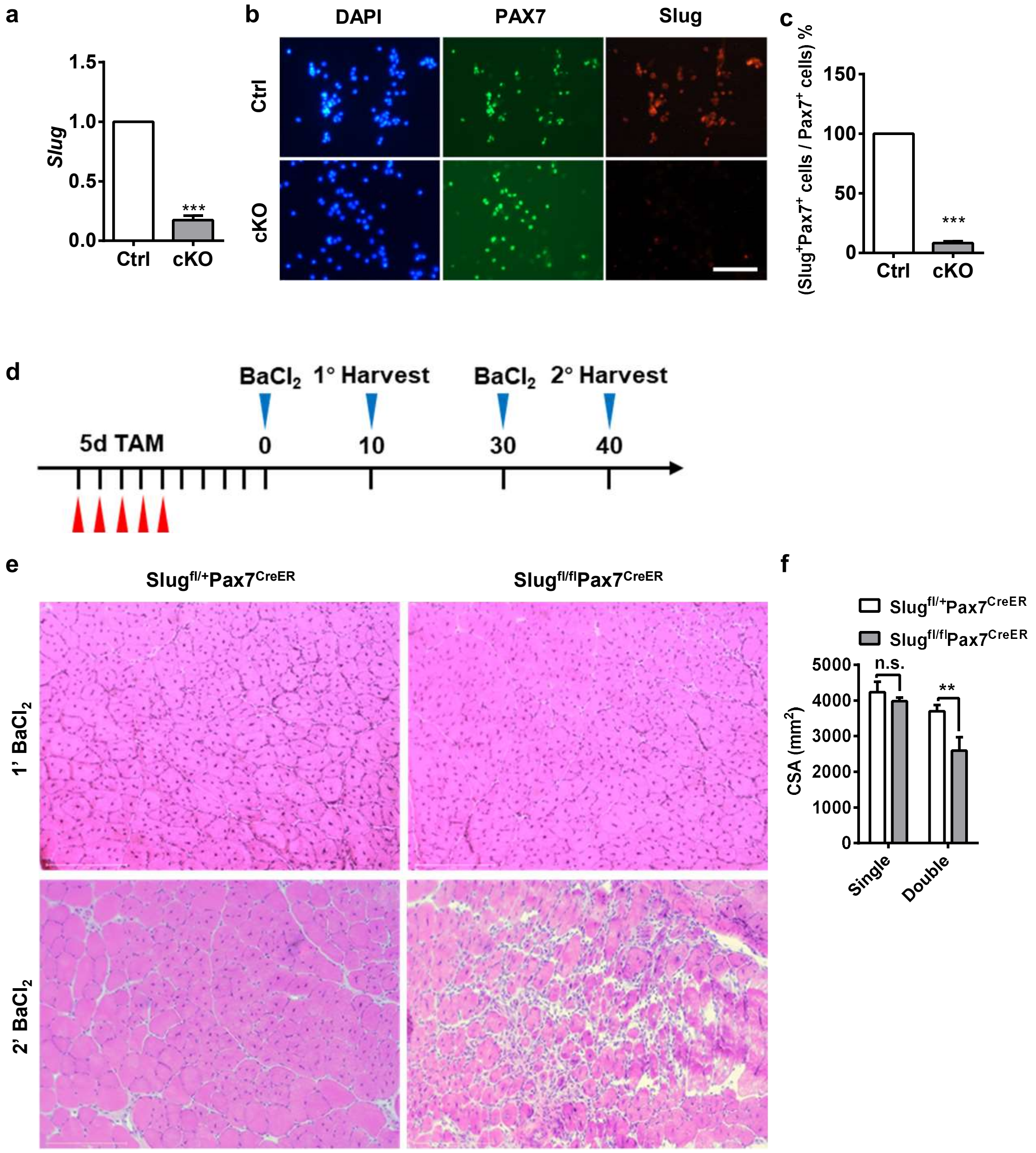

Supplementary Figure 3. Muscle regeneration defect in tamoxifen-induced adult SC-conditional knockout mice.

a. qPCR analysis of tamoxifen-induced Slug deletion in adult SCs (n = 3 mice per group). Ctrl, $Slug^{+/+}Pax7^{CreER}$; cKO, $Slug^{fl/fl}Pax7^{CreER}$. ***$p < 0.001$ by student's *t* test.
b. Immunofluorescence staining of Slug protein in SCs of Ctrl and $Slug^{cKO}$ mice. Scale bar, 200 μm.
c. Quantification of Slug-expressing SCs ($Slug^{+}Pax7^{+}$) as stained in (**b**).
d. Tamoxifen (TAM) regimen, muscle injury and harvest scheme.
e. H&E staining of single and double injured TA muscles in $Slug^{+/+}Pax7^{CreER}$ and $Slug^{fl/fl}Pax7^{CreER}$ mice (n = 5 mice per group). Scale bar, 200 μm.
f. Quantification of the myofiber CSA (mm^2) of single and double injured TA muscles. **$p < 0.01$ by student's *t* test. (n.s., not significant).
Data are shown as mean ± SEM of three independent experiments. Source data are provided as a Source Data file.

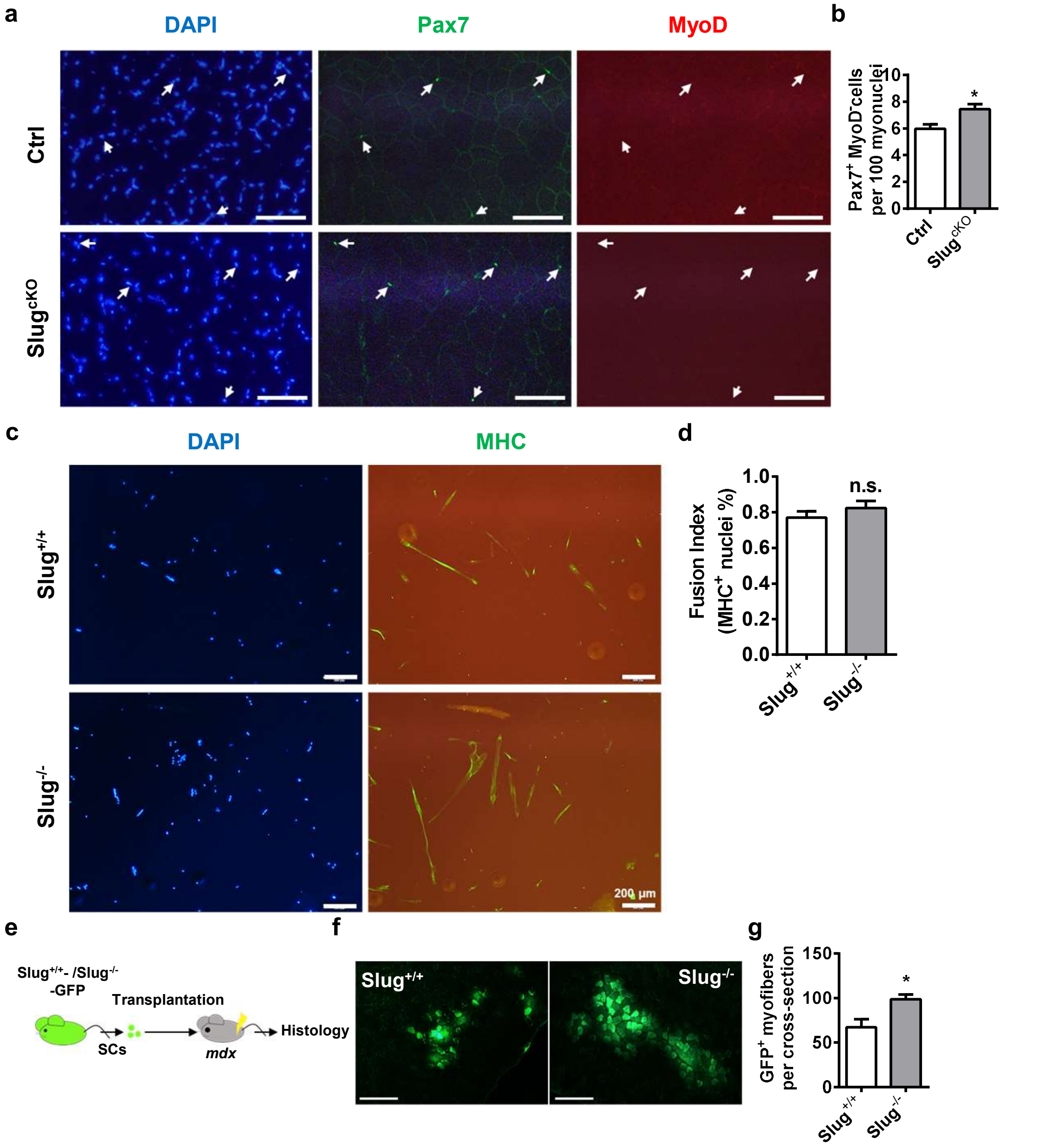

Supplementary Figure 4. Loss of Slug does not impede quiescent SCs from activation and differentiation.

a. Immunohistochemistry of Pax7 and MyoD on transverse sections of resting TA muscles from Ctrl and Slug^{cKO} mice. Scale bar, 100 µm.
b. Quantification of Pax7^{+}MyoD^{-} SCs as stained in (**a**). *p < 0.05 by student's *t* test.
c. Myosin heavy chain (MHC) immunostaining. Primary *Slug*$^{+/+}$ and *Slug*$^{-/-}$ myoblasts were induced for differentiation for 3 days. Scale bar, 200 µm.
d. Quantification of fusion index by calculating percentage of nuclei in MHC^{+} myotubes as stained in (**c**). n.s., not significant.
e. Scheme of SC transplantation. 3000 freshly isolated SCs from *Slug*$^{-/-}$*GFP*-transgenic mice were transplanted immediately into one side of $BaCl_2$-preinjured TA muscles of *mdx* recipients (n = 6 mice). An equal number of SCs from *Slug*$^{+/+}$*GFP*-transgenic mice were injected into contralateral TA muscles of the same recipients as controls. The TA muscles were harvested 4 weeks after transplantation for assessing engrafting efficiency.
f. Representative fluorescent micrographs of GFP-expressing myofibers in frozen TA muscle sections of mdx recipient mice described in (**d**). Scale bar, 100 µm.
g. Quantification of the number of GFP-expressing myofibers in (**f**). *p < 0.05 by student's t test. Data are presented as mean ± SEM of three independent experiments. Source data are provided as a Source Data file.

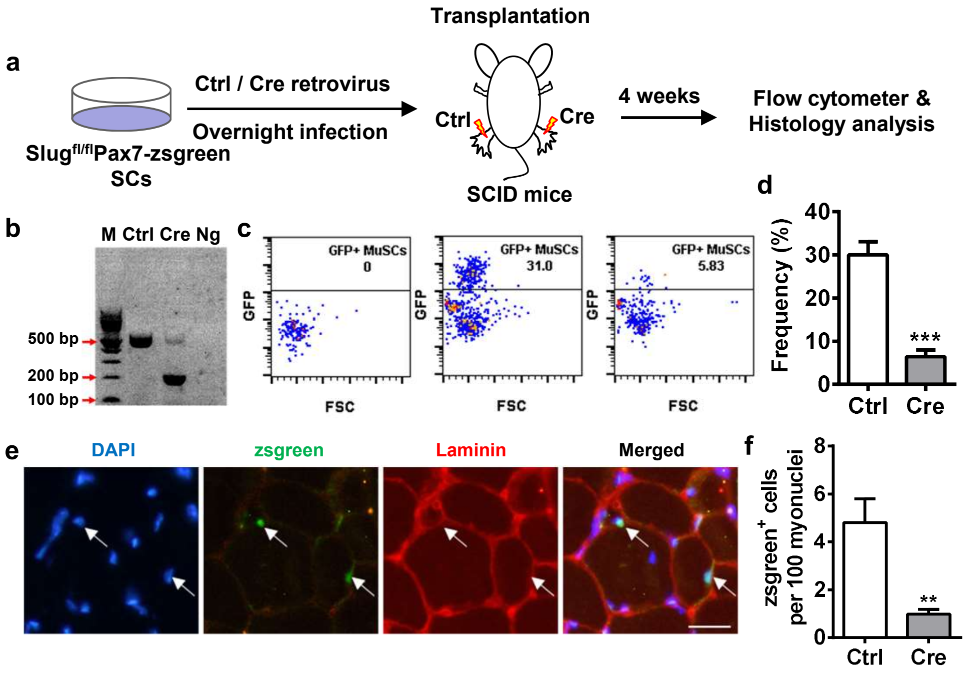

Supplementary Figure 5. Loss of Slug attenuates SC self-renewal capacity.

a. Scheme of *invitro* Slug knockout and transplantation experiment. *Slug*$^{fl/fl}$*Pax7-zsgreen*-derived SCs were infected with lentiviral Cre-recombinase and transplanted into pre-injured TAs of SCID recipients (n = 6 SCID recipients). SCs infected with blank lentiviral vector were injected into the contralateral TAs as control. SC repopulation was analyzed 4 weeks later by flow cytometer and histology analysis.
b. Genotyping PCR confirming viral Cre recombinase-mediated deletion of *Slug* DNA between the two LoxP sequences. PCR products of 530- and 190-bp were expected to be amplified before and after deletion of *Slug* DNA fragment, respectively. M, DNA marker; Ng, negative control for PCR.
c. Representative flow cytometric analysis of the frequency of donor-derived SCs (GFP$^+$) within total recipient MuSCs (CD45$^-$/CD31$^-$/Sca1$^-$/Vcam I$^+$). Muscle cells from TA muscles of untransplanted SCID mice were used as control for setting gate of GFP-negative SCs.
d. Quantification of donor-derived SCs (GFP$^+$) shown in (**c**). ***$p < 0.001$ by student's *t* test.
e. Immunohistochemical analysis of zsgreen and Laminin in recipient muscles transplanted with Ctrl- or Cre-lentivirus-infected SCs. Arrows indicated zsgreen$^+$ cells in satellite cell position. Scale bar, 25 μm.
f. Quantification of donor-derived SCs homing to the satellite cell position after viral infection and transplantation as stained in (**e**). **$p < 0.01$ by student's *t* test.
Data are presented as mean ± SEM of three independent experiments. Source data are provided as a Source Data file.

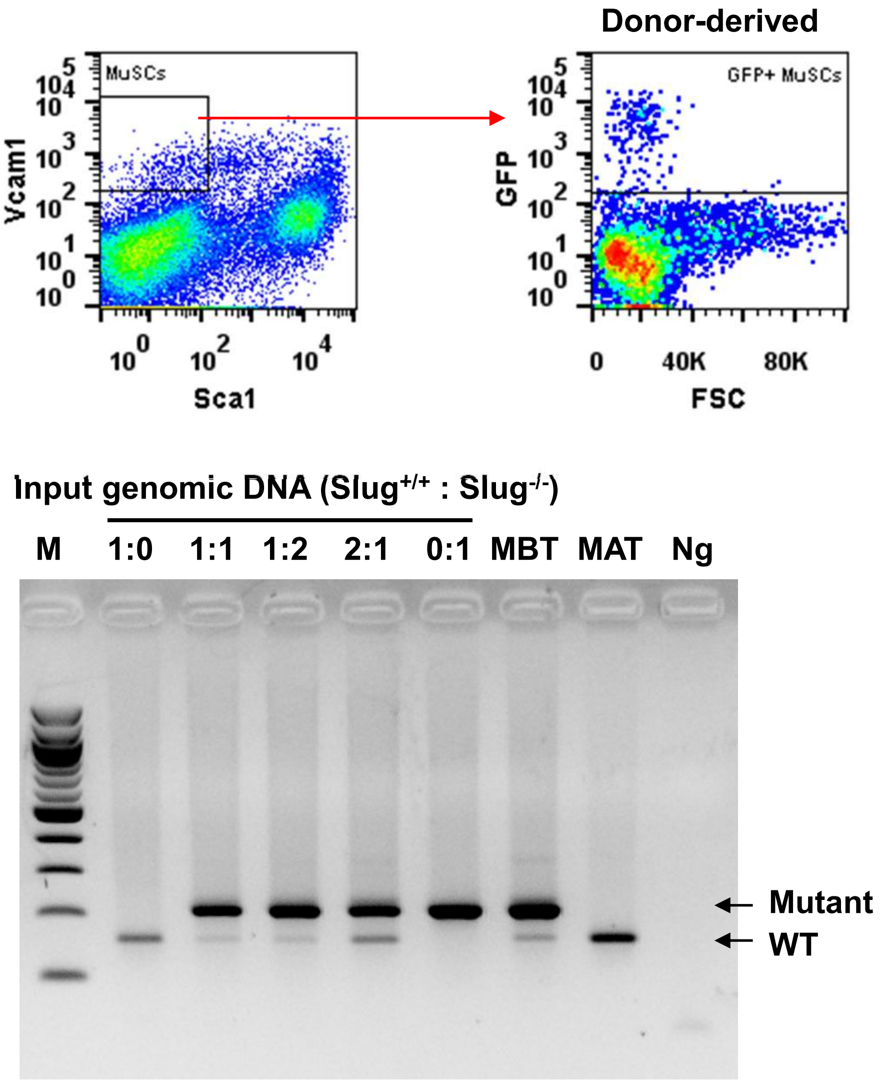

Supplementary Figure 6. An independent repeat of the SC competitive repopulation assay.

SCs from Slug$^{+/+}$GFP- and Slug$^{-/-}$GFP-transgenic mice were mixed at a ratio of 1:1. A small portion of the mixed cells were set aside for extraction of genomic DNA as a control. The remaining cells were then transplanted into pre-injured TA muscles of *mdx* recipients. Four weeks after transplantation, GFP^{+} cells were sorted from recipient MuSCs (CD45^{-}CD31^{-}Sca1^{-}Vcam I^{+}), and subjected to genomic DNA extraction. A series of standard samples by pre-mixing genomic DNAs from each genotype of muscle cells at indicated ratio (Slug$^{+/+}$ vs Slug$^{-/-}$) were used as input for PCR analysis. PCR-products of 157- and 209-bp DNA bands were expected to be amplified from wild-type (WT) and Slug knockout (Mutant) donor-derived genomic DNA, respectively. M, 100-bp DNA ladder; MBT, mixed SCs before transplantation; MAT, mixed SCs after transplantation; Ng, negative PCR control.

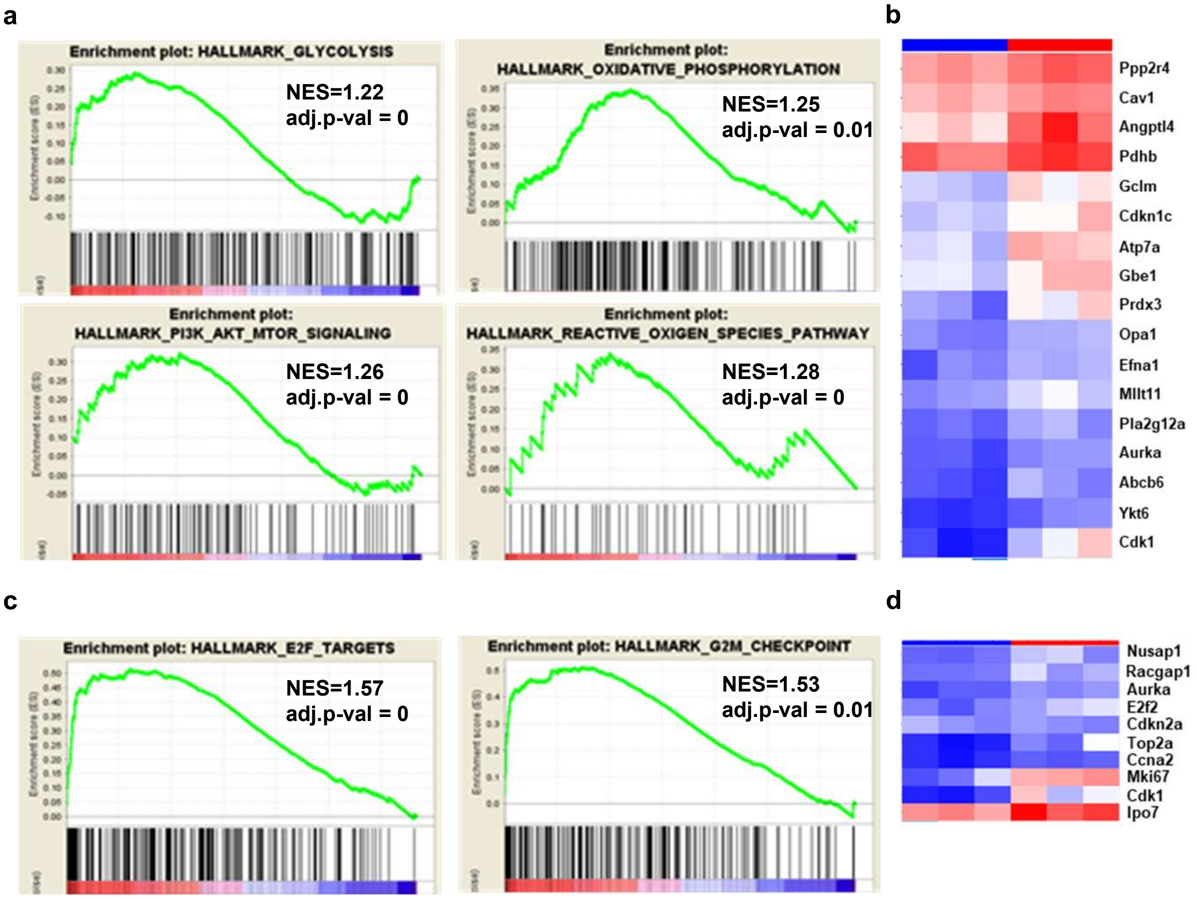

Supplementary Figure 7. altered signaling pathways in Slug-deficient SCs.

a. GSEA enrichment plots for cell metabolism-related pathways.
b. Heatmap showing list of anaerobic and aerobic metabolism pathways-associated genes being up-regulated in Slug-deficient SCs.
c. GSEA enrichment plots for cell cycle-related pathways.
d. Heatmap showing list of cell cycle regulators being up-regulated in Slug-deficient SCs.

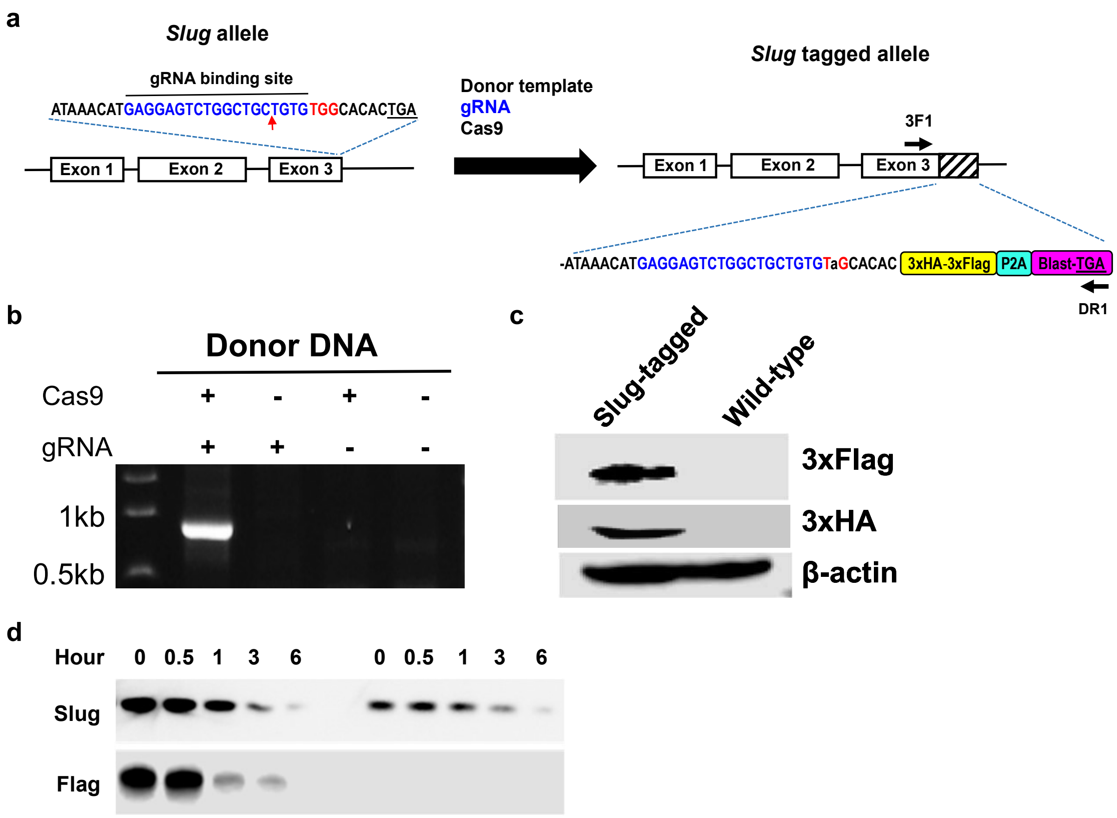

Supplementary Figure 8. Epitope Tagging of endogenous Slug by CRISPR/Cas9-based Genome Editing.

a. Illustrative epitope tagging of endogenous Slug in myoblasts by CRSIPR/Cas9-mediated genome editing.
b. Validation of the correct donor integration. A DNA fragment with an expected size (~900 bp) was amplified only from genomic DNA of myoblasts transfected with the donor plasmid, Cas9 and gRNA.
c. Western blot confirming the correct epitope tagging of Slug.
d. Western blot determining half-life of endogenous Slug protein before and after epitope tagging. Myoblasts were cultured without or with cycloheximide for indicated incubation times.
Source data are provided as a Source Data file.

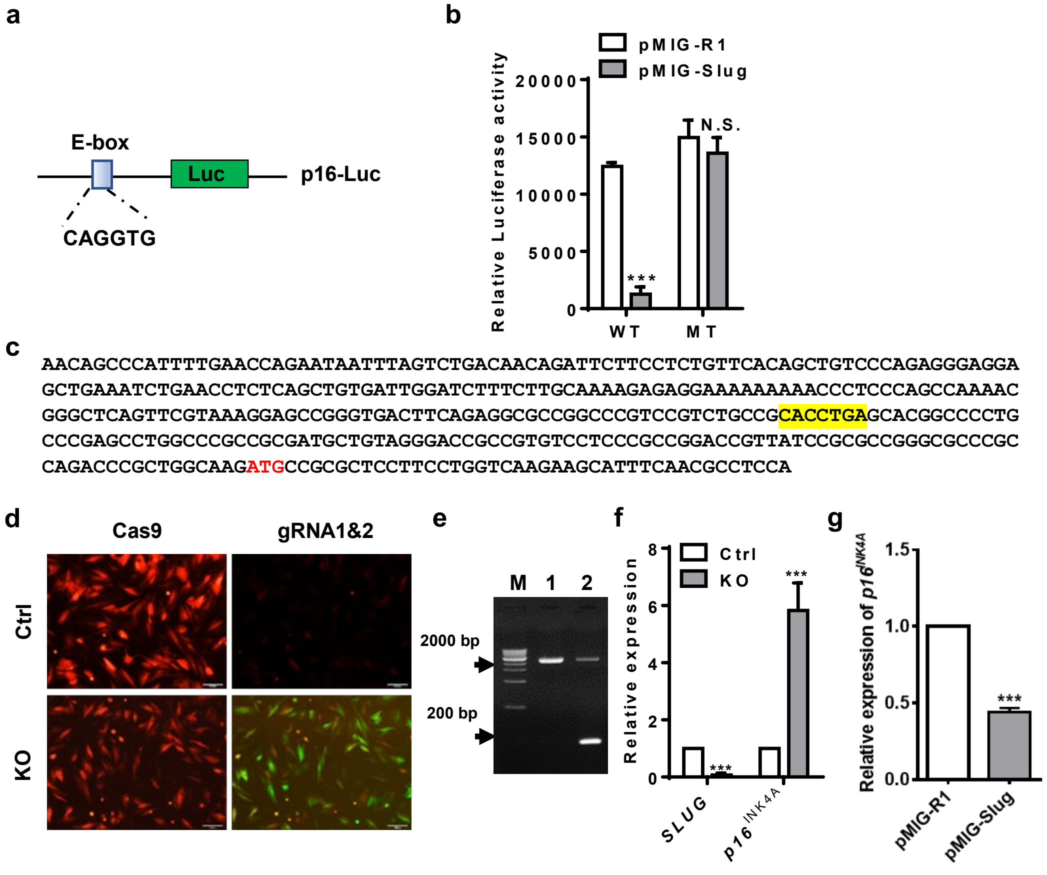

Supplementary Figure 9. Slug directly regulates *p16^Ink4a^* promoter and is conserved in mouse and human.

a. Diagram of the luciferase reporters for the $p16^{Ink4a}$ promoter.
b. Diagram for $p16^{Ink4a}$ luciferase reporter assay.
c. Conserved E-box element (highlighted in yellow) in human $p16^{INK4A}$ promoter. Transcription start coden (ATG) of human $p16^{INK4A}$ was highlighted in red.
d. Representative images showing infection of primary human myoblasts by adenovirus expressing CRISPR/Cas9 (red) and adeno-associated virus expressing two sgRNAs targeting slug sequence (green). Scale bar, 100 µm.
e. Genotyping PCR indicating gRNAs-mediated cutting of *SLUG* genomic DNA by CRISPR/Cas9. 1, AdR-Cas9 infected myoblasts; 2, AdR-Cas9 and AAV-gRNAs infected myoblasts; M, DNA marker.
f. qPCR analysis of $p16^{INK4A}$ expression in Ctrl (Cas9 only) and *SLUG* KO (Cas9 and gRNAs) primary human myoblasts. ***, $p < 0.001$ by student's *t* test.
g. qPCR analysis of $p16^{INK4A}$ expression in control (pMIG-R1) and SLUG overexpressing (pMIG-Slug) primary human myoblasts. ***, $p < 0.001$ by student's *t* test.
Data are shown as mean ± SEM of three independent experiments. Source data are provided as a Source Data file.

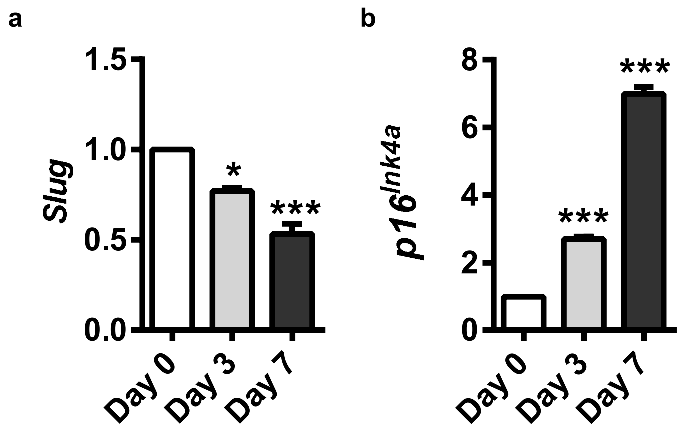

Supplementary Figure 10. Time-dependent expression of *Slug* and *p16*^{Ink4a} in ex-vivo cultured myoblasts.

a. qPCR analysis of *Slug*expression in primary myoblasts on indicated days of culture. $^{*}p < 0.05$, $^{***}p < 0.001$ by student's *t* test.
b. qPCR analysis of *p16*^{Ink4a} in cultured primary myoblasts. $^{***}p < 0.001$ by student's *t* test. Data are shown as mean ± SEM of three independent experiments. Source data are provided as a Source Data file.

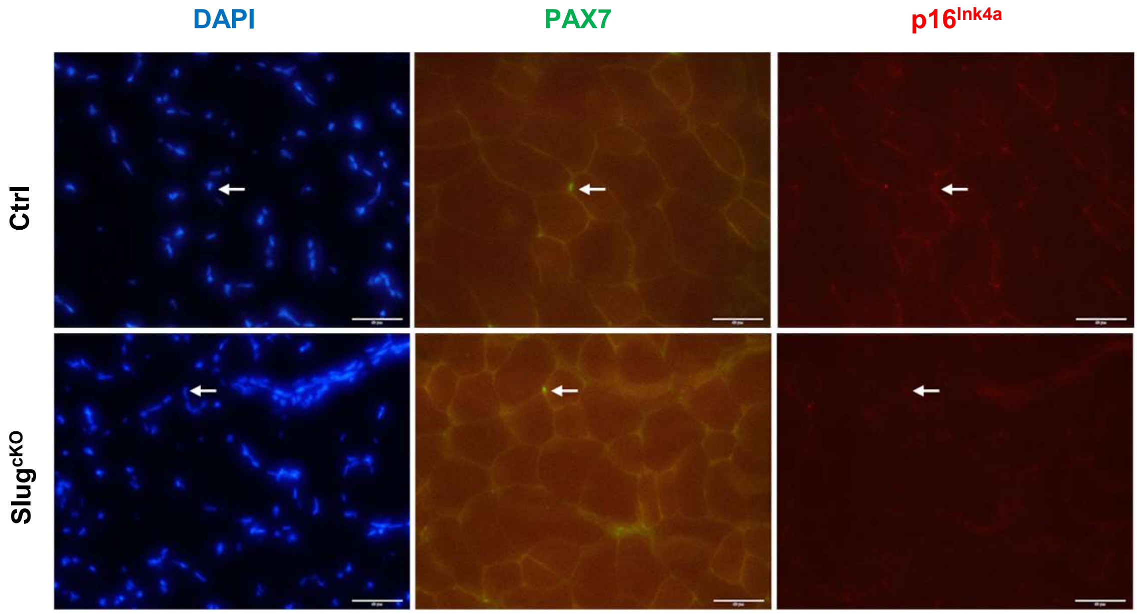

Supplementary Figure 11. Immunohistochemistry for Pax7 and p16^{Ink4a}.

Representative images of IHC co-staining of Pax7 and p16^{Ink4a} protein in SCs within the resting TA muscle of Ctrl and Slug^{cKO} mice (n = 3 mice per genotype). DAPI was used as nuclear counterstaining. White arrows indicate SCs in the section. Scale bar, 50 µm.

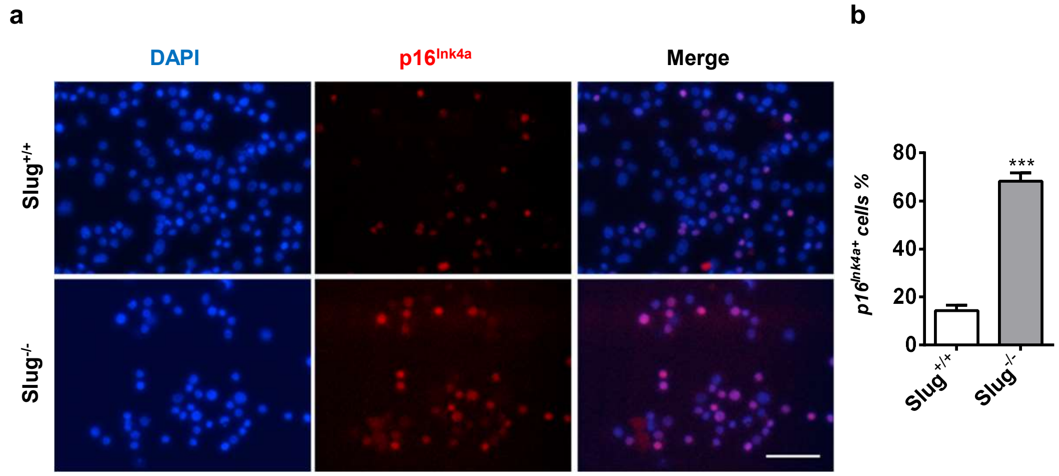

Supplementary Figure 12. p16^{Ink4a} protein expression in *Slug*$^{+/+}$ and *Slug*$^{-/-}$ reserve cell-derived progeny.

a. p16^{Ink4a} immunostaining. Zsgreen^{+} SCs were sorted from hindlimb skeletal muscles of *Slug*$^{+/+}$*Pax7*-zsGreen and *Slug*$^{-/-}$*Pax7*-zsGreen transgenic mice, respectively, and differentiated for 21 days. Confluent myotubes and associated mononucleated cells were then trypsinized and disassociated. Five thousand of sorted zsGreen^{+} reserve cells were re-plated to subculture for another 7 days. p16^{Ink4a} immunostaining was performed on cells at day 7 post culture. Scale bar, 100 μm.
b. Quantification of percentage of p16^{Ink4a} positive cells being stained in (**a**). Data are shown as mean ± SEM of three independent experiments. ***$p < 0.001$ by student's *t* test. Source data are provided as a Source Data file.

a

Slug$^{-/-}$ vs Slug$^{+/+}$ **Old vs Young**

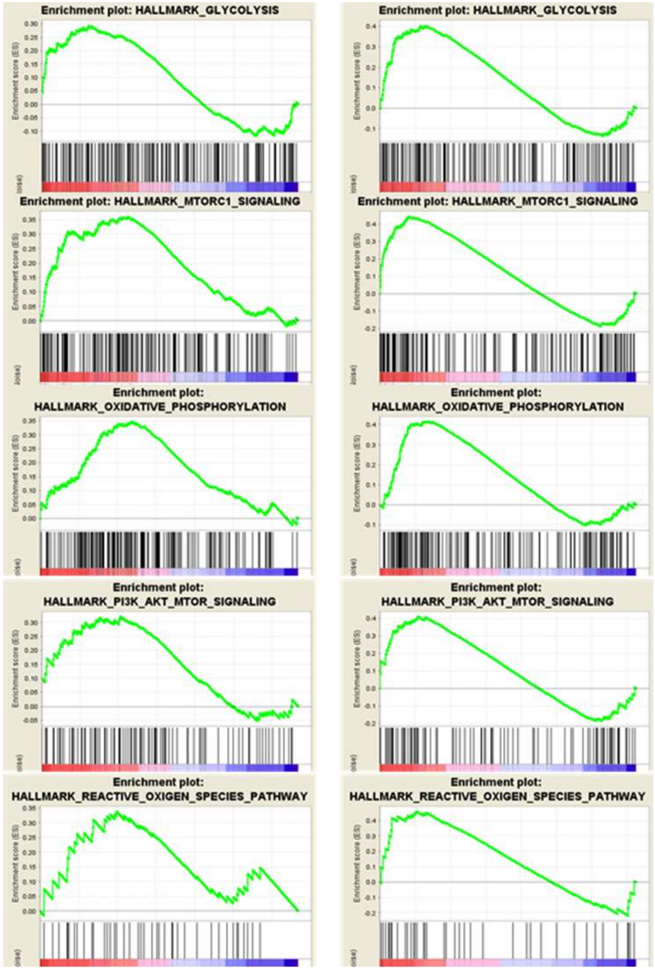

b

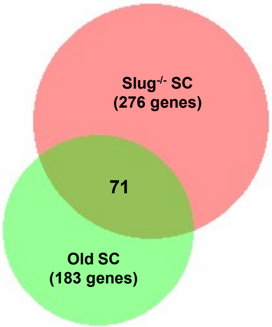

c

Slug$^{-/-}$ vs Slug$^{+/+}$ **Old vs Young**

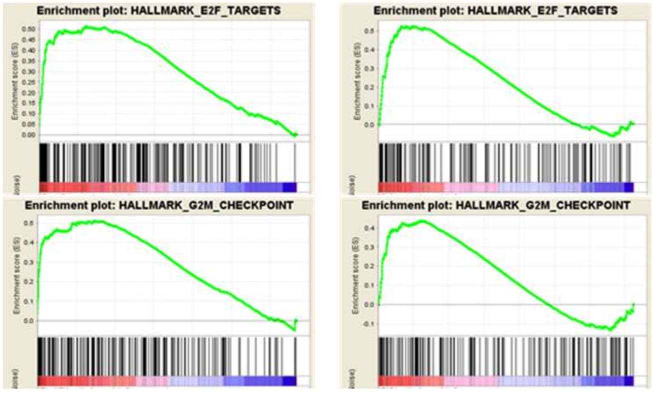

d

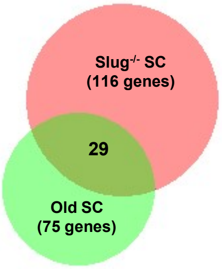

Supplementary Figure 13. Slug$^{-/-}$ SCs resemble aged SCs in altered signaling pathways.

a. GSEA enrichment plots comparing aerobic metabolism pathways activated in *Slug$^{-/-}$* and old SCs.
b. Venn diagram of the overlap between significantly up-regulated genes involved in aerobic metabolism pathways in *Slug$^{-/-}$* and old SCs compared to *Slug$^{+/+}$* and young SCs, respectively.
c. GSEA enrichment plots showing cell cycle regulators induced in *Slug$^{-/-}$* and old SCs.
d. Venn diagram of the overlap between significantly up-regulated genes involved in cell cycle regulators in *Slug$^{-/-}$* and old SCs compared to *Slug$^{+/+}$* and young SCs, respectively.

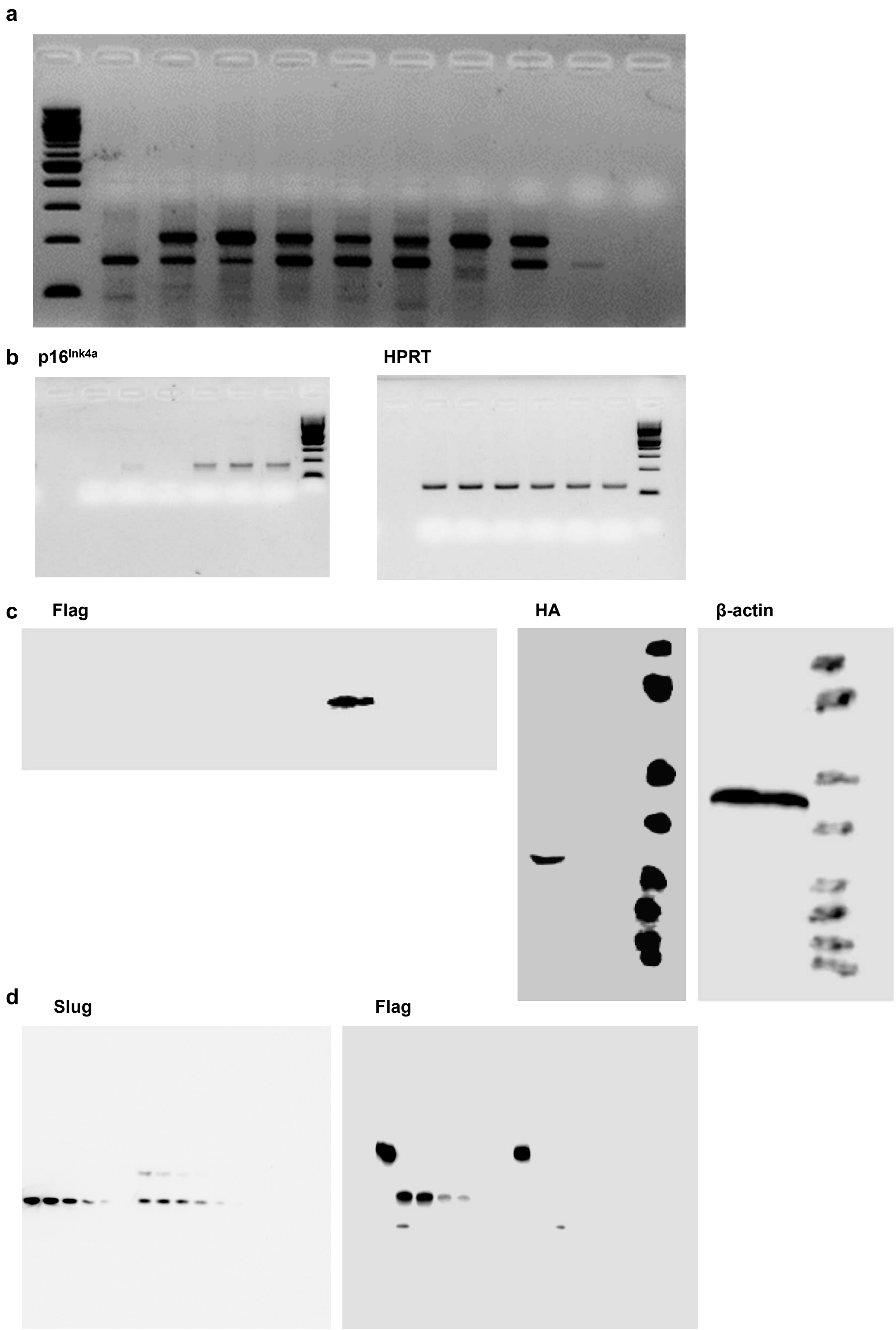

Supplementary Figure 14. Unprocessed protein and DNA gel blots.

a. Original gel imaging for **Fig. 3h**.
b. Original gel imaging for **Fig. 7c**.
c. Original western blot for **Supplementary Figure 8c**.
d. Original western blot for **Supplementary Figure 8d**.

Supplementary Table 1. List of the primers and sequences in the study

Primers	sequences (5'-3')
**Primers for quantitative real-time PCR**	
Slug-qF	GCGAACTGGACACACACACAGTTAT
Slug-qR	GCTGCCGACGATGTCCATACAGTAAT
p16^{Ink4a}-F	ATGGAGTCCGCTGCAGACAGACTG
p16^{Ink4a}-R	CGTTGCCCATCATCATCACCTGAATCGG
p19^{Arf}-qF	GGTTCTTGGTCACTGTGAGGATTCAGCG
p19^{Arf}-qR	TTGCCCATCATCATCACCTGGTCCAGG
HPRT-qF	CTCATGGACTGATTATGGACAGGAC
HPRT-qR	GCAGGTCAGCAAAGAACTTATAGCC
p16^{INK4A}-F (human)	AGCAGCATGGAGCCTTCGG
p16^{INK4A}-R (human)	GCCCATCATCATGACCTGGATCG
GAPDH-F (human)	ATTGACCTCAACTACATGGTTTACATG
GAPDH-R (human)	TTGGAGGGATCTCGCTCCTGGAAG
**Primers for genotyping**	
WTS1 (SlugKO)	TTCTACGTTCTCTGGGCTGG
WTA1 (SlugKO)	GATCTTGCAGACACAAGGCA
KOA2 (SlugKO)	ATTCATCGATGGCCGCTCTA
C015 (p16 KO)	GGCAAATAGCGCCACCTAT
C016 (p16 KO)	GACTCCATGCTGCTCCAGAT
C017 (p16 KO)	GCCGCTGGACCTAATAACTTC
14443 (Cre)	CTCCTCCACATTCCTTGCTC
14444 (Cre)	CGGCCTTCTTCTAGGTTCTG
oIMR1084 (Cre)	GCGGTCTGGCAGTAAAAACTATC
oIMR1085 (Cre)	GTGAAACAGCATTGCTGTCACTT
ProF (Slug^{cKO})	CGTAGGTCACCTAGCGGAAACACGTTTCC
EX-1R (Slug^{cKO})	GTGTGTGTGTCCAGTTCGCTGTAGTTGG
P7B (Slug^{cKO})	CTTCCTGCATATGATTTCATGCTTTGG
